# Why is population information crucial for taxonomy? A case study involving a hybrid swarm and related varieties

**DOI:** 10.1093/aobpla/plw070

**Published:** 2016-11-11

**Authors:** Tobias Marczewski, Yong-Peng Ma, Xue-Mei Zhang, Wei-Bang Sun, A. Jane Marczewski

**Affiliations:** 1 Key Laboratory for Plant Diversity and Biogeography of East Asia, Chinese Academy of Sciences, 132 Lanhei Road, Kunming, Yunnan 650201, China; 2 Yunnan Agricultural University, Kunming, Yunnan 650201, China

**Keywords:** China, hybridization, morphological characters, population variance, *Rhododendron*, taxonomy

## Abstract

Hybridization has become a focal topic in evolutionary biology, and many taxonomists are aware that the process occurs more frequently than previously assumed. Nonetheless many species and varieties are still described without explicitly considering the possibility of hybridization, especially in countries that have relatively short scientific histories, but which often possess the highest species diversities. Furthermore, new taxa are often described based only on herbarium specimens, not taking into account information from wild populations, significantly decreasing the potential to detect morphologies arising from hybridization at this crucial descriptive stage. We used morphological data from a hybrid swarm involving two *Rhododendron* species to showcase possible character combinations in intermediates. Certain characters used to distinguish taxa were more variable within the same individual than between species, emphasizing the importance of population information for an adequate choice of characters. Most described varieties of the two species fell within the spectrum of hybrid morphology, suggesting that these taxa would be unlikely to have merited formal description if contemporary standards had been employed. In all investigated cases the hybrid nature of described varieties seems to have been detectable with adequate morphological data alone, if populations had been assessed. A post hoc assessment of taxa is often complicated, especially if certain types of information are not provided. To avoid accumulation of such invalid taxa, careful scrutiny should be employed for new descriptions. Hybrids (not hybrid species) described as taxa obscure valuable information about natural processes and impact negatively on further research that depends on taxonomic data.

## Abstract (precis)

Despite acceptance in the scientific community that population information and suites of characters are crucial for circumscription of taxonomic groups, new taxa continue to be published on the basis of few herbarium specimens. Given that there is increasing evidence that hybridisation plays an integral part in evolution, it is desirable to identify groups in which it occurs. In this study, we showcase how population variation can be used to identify distinguishing characters and why closely related species that are growing in sympatry should be considered when describing new taxonomic entities.

## Introduction

The occurrence of hybrids, here meaning offspring resulting from two genetically distinct groups of organisms, has been known to taxonomists for a long time (Anderson 1948; Rieseberg 1997). Despite ongoing debate about the overall evolutionary importance of hybridization (Abbott *et al.* 2013), the process has been shown to be widespread and relatively common in plants, occurring across many different families and floras (Ellstrand *et al.* 1996; Whitney *et al.* 2010). Indeed, Rieseberg (1997) suggested that a worldwide average of 11 % of all described plant species may be hybrids, and that this figure may even be an underestimate. Regarding description of hybrids as species, an important distinction should be made between reproductively isolated taxa that originated through hybrid speciation (hybrid species) and continuously re-formed hybrids that have distinct features but do not represent a reproductively isolated lineage (Rieseberg 1997; Hegarty and Hiscock 2005). While there are undoubtedly species of hybrid origin, it is not desirable to attribute taxonomic or species rank to every hybrid.

The distinction of when a hybrid should be described as a potentially new species can be complicated by the circumstance that even significant reproductive isolation is frequently only partial (Rieseberg and Willis 2007). If two only partially reproductively isolated taxa meet in sympatry, hybrids are likely to be formed when habitat conditions allow. Depending on different factors, the realized outcome can vary from only F1s to a hybrid swarm comprising a multitude of different hybrid classes. If backcrosses to one or both parents occur, introgression is generally possible (Anderson and Stebbins 1954), and the parental taxa might show an increased variability. Whether the occurrence of hybrids and/or introgression is merely an artefact of incomplete reproductive isolation (neutral), or is driven by selection, cannot be assessed a priori. Unless evidence suggests an emerging evolutionary lineage that is at least partially reproductively isolated, specific status should not be assigned.

In this paper, we will not further discuss the topic of hybrid speciation or hybrids on the verge of speciation, but will address in more detail issues related to attributing taxonomic status to hybrids that do not fall into these categories. It can often be difficult to determine whether a plant that does not fit any published description is indeed an undescribed species, or a hybrid between two (or more) previously described species (Parnell *et al.* 2013), and over time there have been many examples across different genera and families, where a plant described as a new species has been subsequently proven to be a hybrid (Dowell 1908; Liu *et al.* 2009; Zha *et al.* 2010).

Several problems can arise if a hybrid plant is described as a new taxon. Firstly, it camouflages the actual process occurring in the wild—hybridization—instead suggesting the existence of a genetically distinct, reproductively isolated, entity. A name thus introduced can cause confusion, particularly in the case of species-rich genera, for example, impacting on assessments of species richness, and overall complicating assessments of species distributions. Furthermore, unless hybrids with a particular morphology are common, new taxa described from these few plants may be given a provisional IUCN status of V, E or CR based on the number of individuals, distribution, or number of populations in the wild (IUCN 2014), and could potentially take limited conservation resources from genuine taxa that need them. Additional confusion can arise because, even where a name has been subsequently shown to belong to a hybrid plant, this information can take time to filter into use, and the erroneous name can remain in use long after it should have been abandoned, as relevant literature pertaining to the taxon will still be in circulation and wider use, for example *R. agastum* in the Flora of China (Fang *et al.* 2005) and in the Red List of Rhododendrons (Gibbs *et al.* 2011).

Taxonomic issues arising from hybridization are increasingly being discussed and treated more adequately (Bardy *et al.* 2011; Barrington 2011) However, work on certain floras, especially in species rich regions of the world that have received less attention historically, suggests that problems persist (Parnell *et al.* 2013). Furthermore, a more rigorous taxonomic approach seems to penetrate more slowly into certain species rich genera that receive exceptional interest from outside the scientific community (Pillon and Chase 2007). That these problems still persist could arise from certain taxonomic treatments still being largely based on few herbarium specimens, making it difficult to assess variation apparent across populations, including morphology arising from interspecific geneflow in the wild. Additionally, while hybridization is frequently used to explain patterns in phylogenies, it is often not considered a possibility a priori, and some taxonomists might not be aware of the morphological spectrum potentially visible in hybrids.

There is strong evidence that hybridization does not occur uniformly across all plant families, and hence spontaneous hybridization seems to be more prevalent in certain families and genera (Whitney *et al.* 2010). A group with high levels of natural hybridization is *Rhododendron*, a large genus of woody plants found throughout the northern temperate zone, the Southeast Asian tropics and north-western Australia (Chamberlain *et al.* 1996). The genus has more than 1000 species, many of which are interfertile, and has a centre of diversity in the Sino-Himalayan region (Chamberlain 1982; Milne *et al.* 2010).

That hybridization occurs frequently in *Rhododendron* is well-accepted and extensive evidence of natural hybrid formation can be found across the genus (Kron 1997; Milne *et al.* 2003; Zhang *et al.* 2007; Milne and Abbott 2008; Zha *et al.* 2008; Marczewski *et al.* 2015). Moreover, hybrids can occur between species in different subsections [e.g. *Rhododendron* *delavayi* (subsect. Arborea) and *R. irroratum* (subsect. Irrorata), this study], and have been, more rarely, obtained by artificial pollination even between subgenera (Doorenbos 1955).

Subgenus *Hymenanthes* comprises more than 200 species (Chamberlain 1982) and has high levels of interfertility between even the most divergent species (Milne *et al.* 1999, 2003). A number of described species and varieties in subgenus *Hymenanthes* have subsequently been proven to be hybrids. One example, which we will further investigate in this study, is *Rhododendron* *agastum*, where genetic and morphological data have since demonstrated it to be a hybrid between *R. delavayi* and *R. irroratum* (Zha *et al.* 2010). A further example is *R. flavorufum* [later included as var. *flavorufum* under *R. aganniphum* by Chamberlain (1982)], described on the basis of a split indumentum on the lower lamina surface. Strong evidence from AFLP data identifies this type of indumentum as one phenotype observed in hybrids between *R. aganniphum* and *R. phaeochrysum* (Marczewski *et al.* 2015). The same phenotype (split indumentum) does, however, also occur in hybrid swarms of *R. aganniphum* with other species (D. Chamberlain, personal communication). As the morphological character ‘splitting indumentum’ was explicitly used to delineate this taxon (Chamberlain 1982), potentially leading to different hybrid types being grouped together, a problem was created by failing to correctly assess the actual situation in the natural populations, and thus introducing a taxonomic entity that does not reflect evolutionary relatedness.

At the same time, new species and varieties continue to be described without taking the situation in natural populations into account, as the descriptions are often purely relying on specimen data. The problem is exacerbated by the use of only very few morphological characters to distinguish new taxa from existing ones (e.g. Ming 1984; Chen *et al.* 2010). From the examples given above it can be seen that not only are many species and varieties in *Rhododendron* described on the basis of very few morphological characters, but also that in many cases these subsequently turn out to be hybrid plants. It is our intention, therefore, to examine a natural hybrid swarm in *Rhododendron* and to assess variation in morphological characters found in this swarm. We use this assessment to highlight issues arising from sample size and character choice, as well as to discuss potential morphology in hybrids in the light of later generation hybrids.

The objectives of this paper are therefore (i) to assess the morphological variance in a hybrid swarm of *R. delavayi* and *R. irroratum*; (ii) to relate certain morphological characters used in the descriptions of varieties to hybrid (intermediate) forms; (iii) to discuss whether it is logical to describe hybrid (intermediate) forms as varieties and (iv) to give guidelines for the description of new species/varieties in *Rhododendron* and other genera in which the instance of hybrids is significant.

## Methods

### Study species

**Figure 1:**
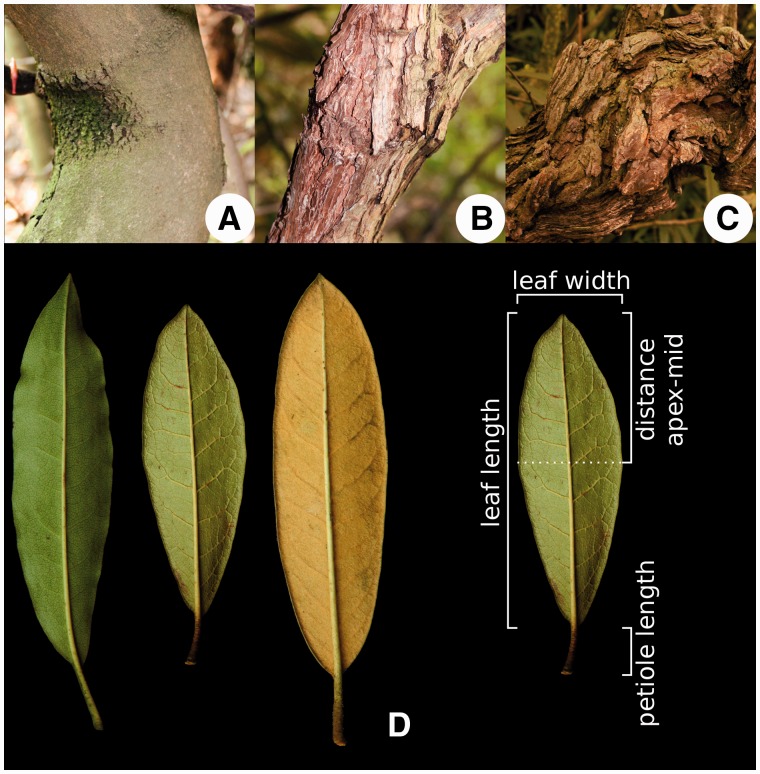
Bark and leaf characters assessed. Different bark types: (A) ‘smooth’, typical for *R. irroratum*, (B) ‘intermediate’, (C) deeply ridged = ‘deep’, typical for *R. delavayi*. (D) Different indumentum types (from left to right): ‘glabrous’/*R. irroratum*, ‘intermediate’, ‘thick’/*R. delavayi*; measured leaf characters.

**Figure 2:**
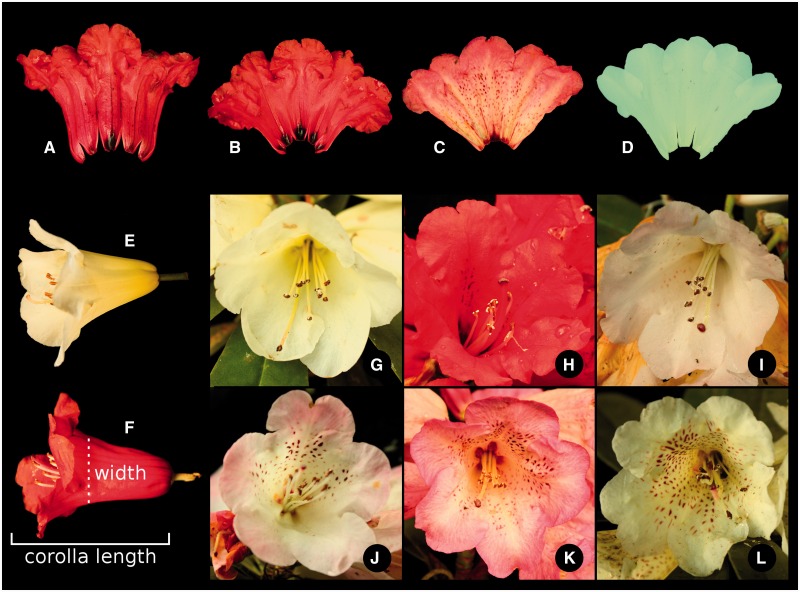
Corolla characters assessed. Nectar pouches and corolla colour: (A) ‘deep-red’ corolla, one ‘dominant’ nectar pouch—the main nectar pouch in the middle is distinctly larger, and has a different colour; (B) ‘deep-red’ corolla, ‘different’ nectar pouches—the middle nectar pouch is perceptibly larger, and the pouches next to the main one are larger then the others, and all are distinctly coloured; (C) ‘pink’ corolla, ‘different’ nectar pouches; (D) ‘cream’ corolla, ‘equal’ nectar pouches—no distinct colouring, and all are the same size. ‘pink-red’ corolla colour is not shown, as it was indistinguishable from ‘deep-red’ in photographs. (E) Typical *R. irroratum* corolla; (F) typical *R. delavayi* corolla, shown are measurements of ‘corolla length’—measured as the length from the calyx to the tip of the main petal (compare A, middle petal), and ‘corolla width’—measured at the top of the corolla tube (where the petals are just still all fused). Maculation: (G) ‘none’—absolutely no markings visible; (H, I) ‘scarce’—some light spots visible on fewer than three petals; (J) ‘few’—spots clearly visible on three petals; (K, L) ‘many’—spots clearly visible on all petals.

**Figure 3:**
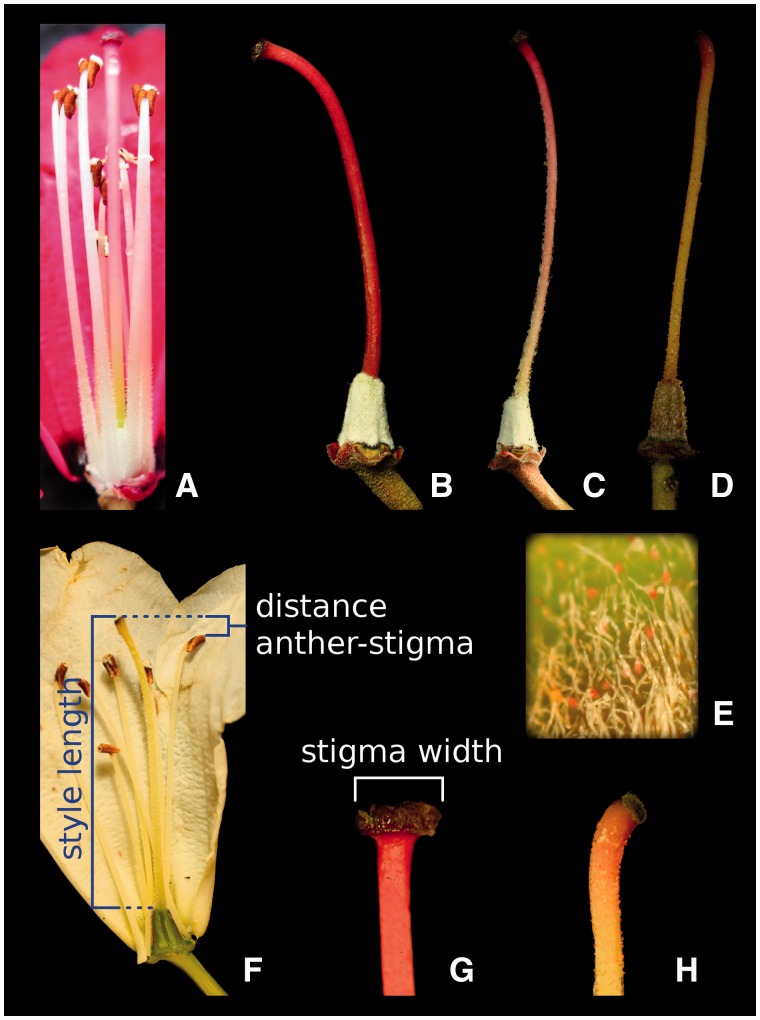
Stamen, style and ovary characters. (A) Hairs on the base of the stamens ‘present’; Style and ovary: (B) ‘red’ style, with no hairs on style—style hairs ‘NA’, ovary hairs are ‘dense’—ovary is not visible through the hairs—and glands are ‘absent’; (C) ‘red-green’ style, that is ‘hairy’, ovary hairs are ‘dense’, and glands are ‘absent’; (D) ‘green’ style, with ‘glandular’ hairs nearly to the top, ovary is ‘hairy’—green ovary visible, and ovary glands are ‘present’. (E) Close-up of the ovary in D, glandular hairs with red heads are clearly visible. (F) Measurements of ‘style length’—from the top of the ovary to the top of the stigma, and ‘distance anther stigma’—the distance from the top of the stigma to the closest anther, when projected onto the style. (G, H) Stigmas; the measurement ‘stigma width’ was always the widest point, as the shape is oval. (B, G) Typical of *R. delavayi*; (D, E, H) typical of *R. irroratum*.

The two study species, *Rhododendron delavayi* and *R. irroratum* are placed in two different subsections, Arborea and Irrorata, respectively, in subgenus *Hymenanthes* (Chamberlain 1982). They co-occur in large parts of their distributions, and are known to hybridise extensively in several sympatric settings (Chamberlain 1982; Zha *et al.* 2010). As could be expected from species in different subsections, they show marked differences in several morphological characteristics (Figs 1–3), enabling relatively easy recognition, but also resulting in hybrids that can exhibit quite different morphologies.

Certain hybrids between *R. delavayi* and *R. irroratum*, probably F1s, were originally described as a species, *R. agastum* Balfour f. & W.W. Smith (Chamberlain 1982). Chamberlain *et al.* (1996) later identified it as a hybrid of *R. delavayi* (as *R. arboreum* ssp. *delavayi*), and suggested *R. decorum* was the other parent. While it is true that an artificial hybrid of these two species does exhibit certain morphological similarities with ‘*R. agastum*’ (Zhang *et al.* 2007; Zha *et* al. 2008), further genetic and morphological work showed convincingly that it is a hybrid of *R. delavayi* and *R. irroratum* (Zha *et al.* 2010). Hence, we can reasonably assume the hybrid status of the intermediate plants in the present study.

*Rhododendron* *decorum* was rare in the study area, and is absent from large parts of it. Furthermore, all hybrids observed showed morphological characters consistent with *R. delavayi *×* R. irroratum* crosses (5 corolla lobes, 10 stamens) rather than those consistent with *R. delavayi *×* R. decorum* hybrids (6+ corolla lobes, 12+ stamens).

### Study area

The area chosen for our study was in the Baili Scenic Reserve in western Guizhou province, China (27.226–27.234N, 105.848–105.856E, altitude: 1600–1700 m), towards the eastern edge of the Yunnan-Guizhou Plateau. The reserve is hilly with coal-rich acidic soil (pH 3–4), and comprises temperate forest dominated by *Rhododendron* species, including both *R. delavayi* and *R. irroratum.* These two species do not show any visible difference in site preference, resulting in almost completely mixed stands, but with locally varying abundance. Furthermore, intermediates are very common, and in certain areas can represent over 50 % of the rhododendron plants. The population seems well established and stable, with the oldest trees likely to be several hundred years old, but all stages from seedlings over young trees to old adults are present. This age structure also applies to the intermediates, with some of them being the tallest trees in the population (∼8 m).

Next to showing a great variability of intermediate forms, the area is easily accessible, as it has been developed for tourism, and is situated close to Yunnan, from where most of the varieties that will be discussed were described; indeed the type specimen for one of the varieties originates from Baili (Chen *et al.* 2010). Due to the proximity of the specimen localities, and occurrence of both species in these areas, it is likely that hybrid morphologies observable in Baili are representative of what might be expected elsewhere.

### Sampling and assessed characters

**Figure 4:**
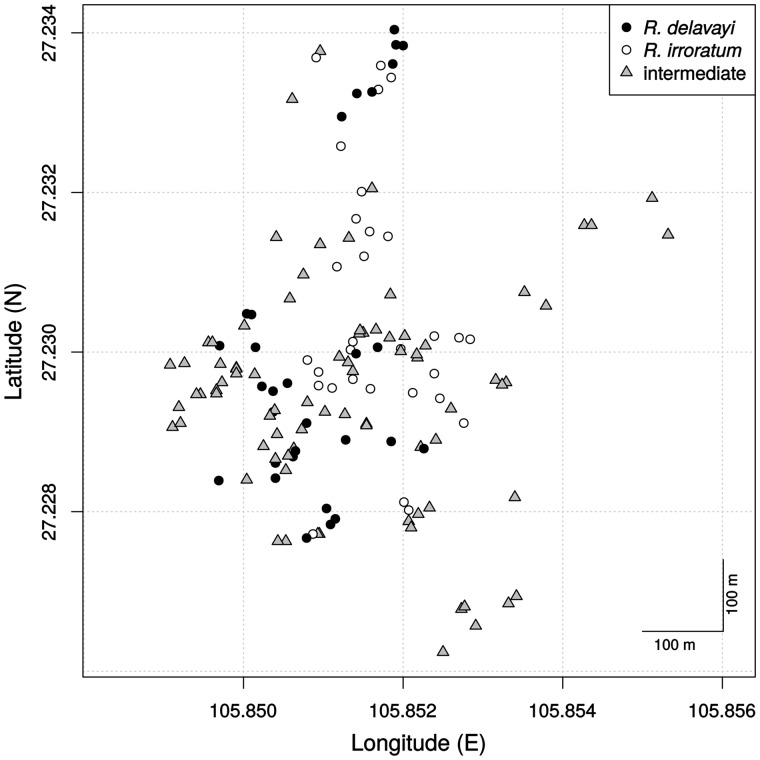
Map of the sampling area in Baili. Indicated are the positions of all individuals that were measured, based on recorded GPS coordinates.

All samples were obtained in Baili in April 2013, during the flowering season of the two species. Altogether 142 plants were assessed, 30 individuals each of *R. delavayi* and *R. irroratum*, and 82 intermediate (hybrid) individuals. Individuals were at this stage assigned to ‘pure’ species based on quick assessment of the characters: flower colour, bark structure, indumentum on lower leaf surface and the presence of hairs on stamens. Most of the intermediate individuals with clearly pink corollas would formerly have been classified as *R. agastum*; and therefore, genetic evidence exists that these individuals are of hybrid origin (Zha *et al.* 2008, 2010). We aimed to cover the whole population, and plants of the same species were sampled at least 10 m apart to avoid kinship, although in some cases intermediate individuals were sampled closer to one individual belonging to one of the two species (Fig. 4). Each day, 10–20 individuals with opened flowers were located, and two to four branches carrying inflorescences were kept for later assessment. The bark of the individual was assessed directly in the field, and for up to 13 inflorescences, if available, the flowers were counted by removing one flower after the other, to avoid double counting. Measurements and qualitative assessment of all other characters was then carried out in a hotel room located close to the population. For measurements on the centimetre scale, rulers were used, and for measurements on the millimetre scale, callipers.

In total we assessed 21 morphological characters (Table 1, 1–21), and for all quantitative characters and some qualitative ones we obtained ten measurements per individual (Table 1, Repeated = yes). From the inflorescences collected, a total of 10 well-preserved flowers were chosen, and 10 leaves were taken to be measured. For the assessment of hairiness, and distinction between normal and glandular hairs 10–20× magnification hand-lenses were employed. For the type of style hairs (Table 1, 21) we also allowed the category ‘NA’, when all styles of one plant were glabrous. The characters measured are illustrated in Figs 1–3. In addition to the quantitative characters directly measured, five derived ratios were calculated (Table 1). Overall leaf size will necessarily have a significant impact on leaf measurements, and to obtain a measure that could be compared between leaves of different sizes the leaf length was used as a general indicator of size, and the other leaf characters were scaled accordingly by dividing through leaf length (Table 1, 22–24). Following the same reasoning the ratio of corolla width to corolla length (Table 1, 25) was calculated, and ‘style hair-cover’ was transformed to the percentage measure of ‘style hair-coverage’ (Table 1, 26).

**Table 1.** Measured characters. Characters that were measured for *R. delavayi*, *R. irroratum* and intermediates. ‘Repeated’ indicates whether several measurements were taken for a single individual (yes = 10 measurements taken per individual).

	Character	Description	Levels/metric unit	Repeated
*Quantitative characters*				
1	Leaf length	Length of the leaf blade	cm	Yes
2	Leaf width	Width of the leaf blade at the widest point	mm	Yes
3	Distance apex-mid	Distance leaf-apex to widest point of leaf	mm	Yes
4	Petiole length	Length of the petiole	mm	Yes
5*	Flowers in inflorescence	Number of flowers in inflorescence	number (count)	Yes
6	Corolla width	Width of corolla at top of tube	cm	Yes
7	Corolla length	Total length of the corolla	cm	Yes
8	Distance anther-stigma	Distance top of anther to top of stigma	cm	Yes
9	Style length	Top of ovary to top of stigma	cm	Yes
10	Style hair-cover	Length of style showing hairs	mm	Yes
11^a^	Stigma width	Stigma width at widest point	mm	Yes
*Qualitative characters*				
12^a^	Bark	Structure of the bark (ridged)	Smooth, intermediate, deep	No
13*	Indumentum	Hairs on the lower leaf surface	Glabrous, intermediate, thick	No
14*	Corolla colour	Colour of the petals	Cream, pink, pink-red, deep-red	No
15*	Nectar pouches	Size and colour of nectar pouches	Equal, different, dominant	No
16	Maculation	Markings on corolla	None, scarce, few, many	No
17^b^	Stamen hairs	Hairs on stamens present or absent	Absent, present	Yes
18*	Ovary hairs	Density of normal hairs on ovary	Hairy, dense	Yes
19*	Ovary glands	Glandular hairs on ovary present?	Absent, present	Yes
20	Style colour	Colour of the style	Green, red-green, red	Yes
21*	Style hairs	Type of hairs on style	Hairy, both, glandular, NA	No
*Derived characters*				
22*	Leaf (width/length)	Leaf width/leaf length	Ratio, no unit	Yes
23*	Leaf (distapex-mid/length)	Distance apex-mid/leaf length	Ratio, no unit	Yes
24*	Leaf (petiole/length)	Petiole length/leaf length	Ratio, no unit	Yes
25*	Corolla (width/length)	Corolla width/corolla length	Ratio, no unit	Yes
26^b^	Style hair-coverage	Style hair cover/style length	Ratio, no unit	Yes

* The character number indicates characters used for both MFA analyses.

^a^Use in MFA 1.

^b^Use in MFA 2 (Fig. 6).

### Type specimens

Type specimens of five infra-specific taxa for the two study species were examined:

(i) For *R. delavayi* var. *adenostylum* Xiang Chen & X. Chen (Y.K. Li 11679, holo HGAS) and *R. delavayi* var. *pilostylum* K.M. Feng (C.W. Wang 87289, holo KM) physical specimens were examined. *R. delavayi* var. *adenostylum* was described on the basis of a glandular style; however, all the styles assessed on the type were glabrous. Because of this, this specimen was included two times in the analyses, once based on observational data and once based on the description.

(ii) For *R. delavayi* var. *peramoenum* (Balf. f. & Forrest) T.L. Ming (Forrest 17708, holo E iso K), *R. irroratum* var. *pogonostylum* (Balf. f. & W.W. Smith) Chamberlain (Henry 11066, holo K iso E) and *R. agastum* Balf. f. & W.W. Smith (Forrest 9920, holo E), measurements were obtained from high-resolution digital images [for more details, **see** **SUPPORTING INFORMATION—Zip Archive]**.

### Statistical analysis

All statistical tests were carried out with the statistical software R, version 3.3.1 (R Development Core Team 2016). For the multiple factor analysis, the package FactoMineR, version 1.33 (Lê et al. 2008) was used, and for the Brown–Forsythe test the package car, version 2.1-0, which provides the function leveneTest (corresponding to the Brown–Forsythe test when setting centre = median).

***Outliers, normality and variance of the data.*** These data were checked for outliers with Cleaveland plots. Deviation of the quantitative data from a normal distribution was assessed with a Shapiro–Wilk test, and equality of variances was checked by comparing the range of variances and using Brown–Forsythe tests. Variances and normality were checked between individuals for repeated measurements, and for means of individuals between species. For repeated quantitative characters, the means for individuals were calculated as trimmed means to reduce the impact of extreme values; this was not done for qualitative characters with repeated measurements, as they are often by definition extreme (e.g. 0 or 1).

***Multiple factor analysis* (*MFA*).** To reduce noise levels in the data, characters that were not well-represented in the first two dimensions were excluded after running an exploratory MFA (**see** **SUPPORTING INFORMATION—PDF**). For all analysis the option of scaling quantitative characters to unit variance was used. Two MFAs were subsequently performed:

MFA (1) included only individuals from Baili, and used 13 characters, excluding ‘stamen hairs’ and ‘style hair-coverage’ (Table 1). This was performed to calculate a hybrid index for each individual and test for correlation of this index with the characters ‘stamen hairs’ and ‘style hair-coverage’ (see following paragraphs).

MFA (2) combined data from Baili individuals with type specimens, based on 13 characters, excluding ‘bark’ (no data available for type specimens) and ‘stigma width’ (distorted in dried material) (Table 1).

***Re-assignment of individuals to species*.** The first two co-ordinates of MFA 1 were used to re-define individuals belonging to one of the two parental species in the following manner. Firstly, the initial species assignment was used as a prior, and the thus grouped individuals were used to calculate the species means along both axes, and their corresponding standard deviation. Secondly, a 99 % confidence interval around the means on both axes was used to designate the co-ordinate ranges in which individuals were considered to be ‘pure’ members of one of the species. All individuals were then re-assigned according to their co-ordinates. If they fell into the confidence limits of one of the species they were assigned to this species, and if they fell outside, they were designated intermediate. All analyses were then carried out using this adjusted species assignment.

***Hybrid index*.** One of the objectives of this study was to assess how the two morphological characters ‘stamen hairs’ and ‘style hair-coverage’ behave with regards to a mixed genetic background. Therefore, it was desirable to assign an index of hybridity to each intermediate individual. As variance of the two characters was represented in the first dimension of MFA 1, but not in the second dimension, we used the co-ordinates of individuals along the first axis to devise a hybrid index. The means of the two parental species were chosen as end points of this index; because the two means were not exactly mirrored with respect to 0 [**see** **SUPPORTING INFORMATION**—**Figure S1**], the average of the two means were used as an adjustment to retain symmetry. For ease of use the index was then scaled to fall between 0 and 1, where 0 corresponded to the adjusted mean of *R. irroratum* and 1 to the adjusted mean of *R. delavayi*.

***Correlation of morphological characters with the hybrid*** ***index*.** The morphological characters ‘stamen hairs’ and ‘style hair-coverage’ were then tested for correlation with the above calculated hybrid index. The bounded response variable ‘style hair-coverage’ (percentage) was converted to a categorical variable with four categories (corresponding value): ‘no hairs’ (0); ‘hairs in lower half’ (<0.5); ‘hairs in upper half’ (>0.5); ‘hairs to top’ (1). Potential correlation with the hybrid index was then assessed using Kendall's rank correlation; once for all individuals, and, to reduce the influence of the *R. irroratum* individuals, once for individuals with a hybrid index above 0.5, meaning a gradient from *R. delavayi* to the centre of the intermediates. For the binary response variable ‘stamen hairs’ the point biserial correlation coefficient (= Pearson product–moment correlation) was calculated; once for all individuals, and once for only the intermediates (excluding both parental species).

***ANOVA*.** For the quantitative characters and derived ratios, the variances of repeated measurements of individuals showed a very wide range, and were significantly different. This means that the significance of the *F*-values resulting from an ANOVA could not be interpreted. However, a hierarchical ANOVA still provides an overview of how much different levels contribute to the overall variance. Hence, the reason to carry out an ANOVA was mainly to investigate if certain characters showed a variance contribution added by the level of species that exceeded the variance already explained by the variance found within species or even within individuals. Thus, the contribution of the different levels: ‘differences between measurements of the same individual’, ‘differences between measurements of individuals of the same species’ and ‘differences between species’ was used as an indicator of whether the character in question had the general power to distinguish species, or if the variation between individuals or even within one individual exceeded species differences, thus not meriting the use of the character for species discrimination. For this analysis only the individuals attributed to one of the two parental species were included, as the variance of the intermediates was, as can be expected, significantly higher than either.

To obtain the variance contribution, a hierarchical ANOVA was performed and the returned sums of squared differences (ssd) for each level were adjusted for sample size differences, and their respective contribution to the total was then calculated following Cockerham (1973) [also **see** **SUPPORTING INFORMATION—Zip Archive**].

## Results

### Excluded individuals

In both species, the style is generally longer than the anthers, and this distance was recorded as a positive number. However, in four individuals (one designated as *R. delavayi* and three intermediate), the style was significantly shorter than the anther, resulting in a ‘negative’ distance. Furthermore, this resulted in a bi-modal distribution of style–length for the data. These individuals were considered aberrant, and as one of the characters to which we wanted to pay special attention, ‘style hair-coverage’, may well be closely linked to the style length, these four individuals were completely removed from the dataset. A further individual classified as intermediate showed exceptionally large leaves, which might have interfered with its placement in the MFA, and we therefore decided to remove it from the analysis. Hence, in total five individuals were removed from the dataset, leaving 137 included plants.

### Character measurements

**Figure 5:**
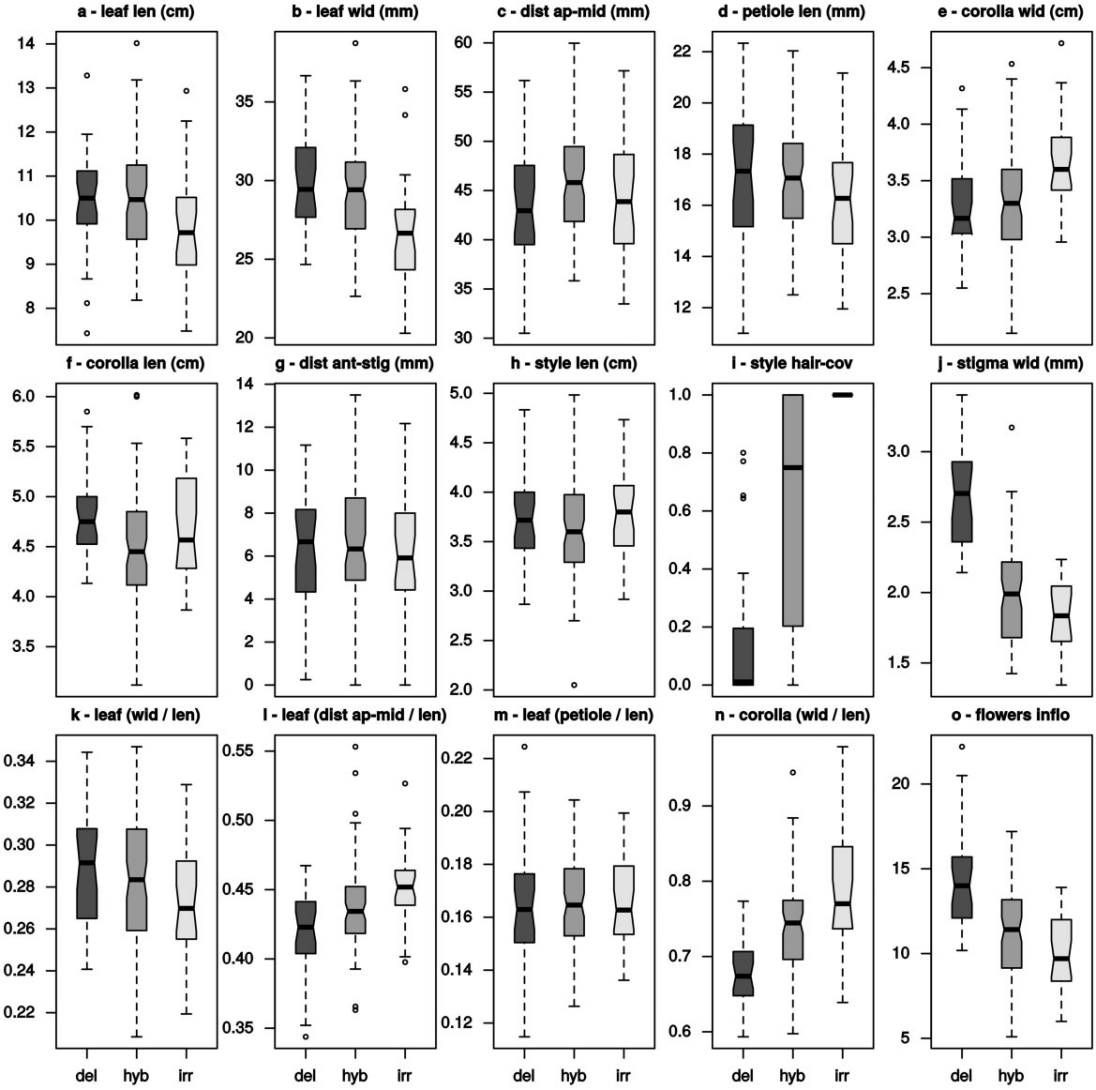
Ranges of means for the quantitative characters measured. Boxplots showing the ranges of observed means (trimmed means, calculated from repeated measurements of the same individual) for the measured quantitative characters and derived ratios of the two parental species *R. delavayi* (del) and *R. irroratum* (irr) and intermediates (hyb). Horizontal black lines indicate the median and notches the 95 % quartiles, hence non-overlapping notches can be interpreted as significant difference. Notches are not shown for style hair-coverage (i), as the distribution of this percentage measure is too skewed in the parental species.

Apart from a few exceptions, the variances of repeated measurements for individuals of the same group were significantly different from each other [**see** **SUPPORTING INFORMATION—Table S1**]. This might have been due to the small sample size (10 measurements per individual), combined with the large range that most characters showed (Table 2, range). Furthermore, the distributions of the measurements within groups did not fit a normal distribution, as determined by a Shapiro–Wilk test. According to the central limit theorem the means of the individuals should, however, be normally distributed. After reducing the impact of extreme values by calculating trimmed means (R function mean with trim = 0.2), the distributions of individual means did not significantly deviate from a normal distribution, apart from the character ‘corolla length’ in *R. irroratum* (Shapiro–Wilk, *P* = 0.05), and ‘leaf (dist apex-mid/length)’ in the intermediates (*P* < 0.01). These trimmed means were used for analyses, apart from the ANOVA. Ranges of the trimmed means are given in Fig. 5.

**Table 2.** Range of quantitative characters. Ranges of measured quantitative characters, within the three groups (*R. delavayi*, *R. irroratum* and intermediates). Shown are the ranges for all measurements; the range of the trimmed individual means (calculated for each individual from six measurements, after trimming the two lowest and highest) and the overall mean. The <0 for distance anthers-stigma (8) indicates that some negative measurements (style shorter than anthers) were observed, but individuals with consistently shorter styles were removed from the data.

Character		*R. delavayi*			Intermediates			*R. irroratum*		
		Range	Range (means)	Overall mean	Range	Range (means)	Overall mean	Range	Range (means)	Overall mean
1	Leaf length	4.50–18.40	7.43–13.28	10.19	3.90–21.90	8.18–14.02	10.35	4.40–16.50	7.48–12.93	9.8
2	Leaf width	9.00–51.00	24.67–36.67	29.43	8.60–50.00	22.64–38.73	29.05	11.25–44.00	20.30–35.82	26.55
3	Distance apex-mid	15.38–78.25	30.50–56.18	42.99	11.34–79.04	35.83–59.97	45.43	18.73–76.00	33.48–57.17	44.31
4	Petiole length	6.00–31.00	11.00–22.33	17.04	5.00–35.00	12.50–22.03	17.16	6.53–27.34	11.95–21.17	16.13
5	Flowers in inflorescence	5.00–25.00	10.18–22.20	14.15	2.00–22.00	5.09–17.20	11.34	4.00–17.00	6.00–13.90	10.25
6	Corolla width	1.80–4.70	2.55–4.32	3.29	1.70–5.20	2.15–4.53	3.31	2.30–5.20	2.96–4.72	3.66
7	Corolla length	3.20–6.20	4.13–5.85	4.79	2.00–6.40	3.12–6.02	4.45	3.00–6.10	3.87–5.58	4.66
8	Distance anther-stigma	<0–15.00	0.25–11.17	6.29	<0–26.00	0–13.50	6.61	0–14.00	0–12.17	6.36
9	Style length	1.90–5.00	2.87–4.83	3.72	1.40–5.50	2.05–4.98	3.62	2.30–5.00	2.92–4.73	3.78
10	Stigma width	1.52–4.13	2.14–3.40	2.65	1.00–3.43	1.42–3.17	2.02	1.00–2.60	1.34–2.23	1.82
11	Leaf (width/length)	0.14–0.45	0.24–0.34	0.29	0.08–0.47	0.21–0.35	0.28	0.15–0.45	0.22–0.33	0.27
12	Leaf (dist apex-mid/length)	0.24–0.63	0.34–0.47	0.42	0.13–0.65	0.36–0.55	0.44	0.34–0.60	0.40–0.53	0.45
13	Leaf (petiole/length)	0.09–0.29	0.11–0.22	0.17	0.06–0.28	0.13–0.20	0.17	0.11–0.26	0.14–0.20	0.17
14	Corolla (width/length)	0.50–1.09	0.59–0.77	0.69	0.46–1.39	0.60–0.94	0.75	0.55–1.17	0.64–0.98	0.79

## MFA 1

MFA 1 resulted in a clear separation of the two parental groups along the first axis [**see** **SUPPORTING INFORMATION—Figure S1a**]. Inspection of a plot of the eigenvalues indicated that the data are reasonably well represented by the first two dimensions [**see** **SUPPORTING INFORMATION—Figure S2**], with the first dimension explaining 28.55 % and the second 17.55 % of the variation. The second dimension seemed mostly to capture heterozygosity (or intermediacy) and was therefore also deemed useful for the re-assignment of individuals to species.

### Re-assignment of individuals to species

Using the initial species assignments as prior information, *R. delavayi* individuals had a mean (±sd) of 3.22 (±0.36) for the first dimension, and 1.74 (±0.44) for the second; *R. irroratum(Color online) * individuals had means of −3.15 (±0.39), and 1.77 (±0.19), respectively. Using 99 % confidence intervals around those means to define the species resulted in three individuals being re-assigned: one individual previously classified as *R. delavayi* was changed to intermediate, one intermediate individual satisfied the condition for *R. delavayi*, and one *R. irroratum* individual was re-classified as intermediate. Hence, using the final classification, of the 137 individuals, 29 were classified as *R. delavayi*, 29 as *R. irroratum* and 79 as intermediate. These classifications were henceforth used throughout the present study.

### Correlation of style and stamen hair-cover with hybrid index

**Figure 6:**
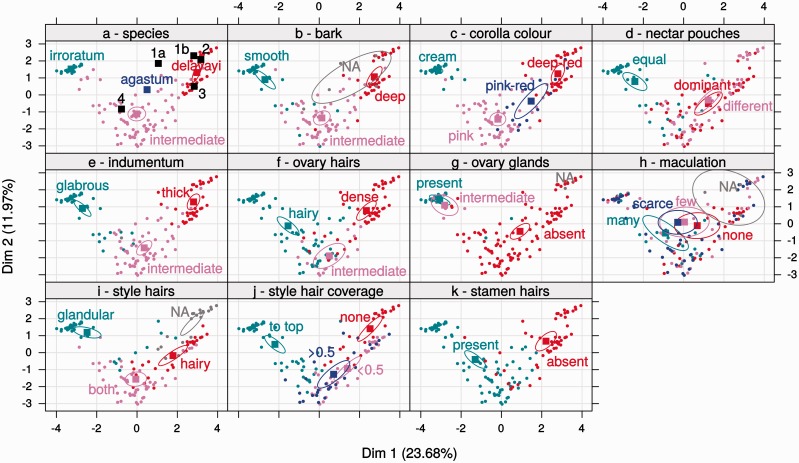
Multiple Factor Analysis (MFA 2) using combined data from Baili and type specimens. The following characters shown here were not used to calculate the MFA and were only plotted onto the coordinates for graphical assessment: species (a), bark (b) and maculation (h). (a) Species groupings and placement of assessed type specimens: 1a—*R. delavayi* var. *adenostylum* (according to description); 1b—*R. delavayi* var. *adenostylum* (based on characters seen on specimen); 2—*R. delavayi* var. *peramoenum*; 3—*R. delavayi* var. *pilostylum*; 4—*R. irroratum* subsp. *pogonostylum* and agastum—‘*R. agastum*’.

Using the co-ordinates along the first MFA dimension to calculate a hybrid index, intermediates showed values in the range of 0.03–0.88, where an index of 0 would indicate the mean of *R. irroratum* and 1 the mean of *R. delavayi*, while individuals of *R. irroratum* had values in the range of –0.13 to 0.10, and individuals of *R. delavayi* of 0.92–1.13. The correlation of ‘style hair-coverage’ with this hybrid index was highly significant using all individuals (Kendall's tau = −0.62, *z* = −10.109, *P* < 0.001), and remained significant when only looking at the range from *R. delavayi* to the middle of the intermediates (hybrid index >0.5, compare Fig. 6j), however, the correlation was less pronounced (Kendall's tau = −0.33, *z* = −3.705, *P* < 0.001). The same significant correlation with the hybrid index was observed for the presence of ‘stamen hairs’ (Fig. 6k) when using all individuals (Pearson's *r* = −0.71, *t* = −11.801, df = 135, *P* < 0.001), which remained significant when only looking at a correlation in all intermediates (Pearson's *r* = −0.33, *t* = −3.069, df = 77, *P* = 0.003).

## ANOVA

As explained before, significance of the hierarchical ANOVA could not be directly assessed, due to large variance differences between measurements of single individuals. This also probably influenced the variation attributed to the different levels, which in several cases led to a negative contribution of the level ‘between species’. An additional explanation for these negative values is that the top-most level is not represented in the data of the respective character, and as variation is already fully explained by the two lower levels, assuming a further level introduces an artefact. Hence, we interpreted a negative value for ‘between species’ as meaning that the character in question does in fact not discriminate between species.

Using this criterion leaf characters were generally bad at species discrimination (Table 3, 1–4, Fig. 5), showing most variation attributed to differences of measurements of the same individual. Even ‘leaf width’, which seemed to tend to be different between the species, was only marginally so. The size-corrected ratios of leaf characters (Table 3, 11–13) showed less variation within individuals, but two of them (11, 13) were still not different between species. Only the size corrected ‘distance from apex of leaf to widest point’ was indicated as having some discriminatory element to it. While the flower measures also showed doubtful discriminatory power (Table 3, 6, 7), their ratio was indicated to be different enough to distinguish between species (Table 3, 14). Also the number of flowers per inflorescence showed a marked variation contribution from ‘between species’ (Table 3, 5). The style related characters ‘style length’ and ‘distance from anther to stigma’ were more homogeneous within individuals, but differences between individuals of the same species were still much larger than differences between species (Table 3, 8, 9). The quantitative character showing the highest discrimination between species was ‘stigma width’ (Table 3, 10).

**Table 3.** Contribution to observed variation in quantitative measurements. Results from a hierarchical ANOVA using the data from the two parental species *R. delavayi* and *R. irroratum*; intermediates were not included. Variation in quantitative measurements explained by differences between measurements: within the same individual, between individuals of the same species and between species. Negative values for the level ‘between species’ indicate that this level is not present in the data, which results in an artefact. Characters with large contribution from differences between species are marked with **, and the ones for which the level seems to be somehow meaningful are marked with *.

	Character		% variation explained		
			Between species	ind w/species	w/individuals
1	Leaf length		−10.6	25.8	84.8
2	Leaf width	*	1.2	12.6	86.1
3	Distance apex-mid		−11.1	25.1	86
4	Petiole length		−10	27.1	82.9
5	Flowers in inflorescence	**	29.5	32.3	38.3
6	Corolla width	*	3.6	47	49.5
7	Corolla length		−23.4	71.9	51.5
8	Distance anther-stigma		−24.9	67.6	57.3
9	Style length		−30.6	87.5	43.1
10	stigma width	**	61	24.9	14.1
11	Leaf (width/length)		−4.5	38.9	65.6
12	Leaf (dist apex-mid/length)	*	7.8	30.2	62
13	leaf (petiole/length)		−19.9	50.8	69.1
14	Corolla (width/length)	**	33.2	32.9	33.9

## MFA 2

For MFA 2, which used a slightly different character set, the first and second axes accounted for 23.68 and 11.97 % of the variation, respectively; however, this did not alter the overall grouping patterns (Fig. 6a). All of the described varieties of *R. delavayi* were placed close to *R. delavayi* individuals from Baili (Fig. 6a, 1–3); *R. irroratum* subsp. *pogonostylum* and ‘*R. agastum’*grouped with the intermediates (Fig. 6a, 4, agastum).

## Discussion

### Morphological characters

Because the two study species were not very closely related, many of the qualitative characters were well distinguished (Fig. 6b–g, i). This resulted in the initial assignment of individuals to species being relatively congruent with later assessment, taking the MFA results into account. However, even though the initial assessment was carried out by scientists familiar with the species and aware of the presence of hybrids, three individuals were doubtfully assigned, as they either did not fulfil the species requirements, or were classed as intermediate despite fulfilling them. These errors occurred at the margins of the later defined species confidence intervals, and with the present data there can be, of course, no certainty of the correctness of the groups defined later. Additionally, due to the limited number of categories, qualitative characters can sometimes easily be interpreted differently depending on several factors, including age of flower, plants that were investigated directly before the one assessed, or just time of day and thus light quality. Generally this should not affect many characters, but if few characters only are used to delimit a group of individuals, difference in one character might overestimate the uniqueness of a single individual.

Further confusion can arise from using an easily visible character that is, without assessment, apparently characteristic. In the descriptions of *R. irroratum* and ‘*R. agastum*’ the presence of spots on petals is explicitly mentioned, while there is no such mention for *R. delavayi* (Chamberlain 1982). This suggests a difference which cannot be maintained using the current data, as maculation is not correlated with any grouping (Fig. 6h). A possible explanation is that dark spots are necessarily more visible on a lighter corolla (white or pink) than on deep-red petals, and might not be at all visible if a dark corolla has been dried, as on a herbarium specimen. Hence, knowledge of the species in the field, including variation in a population, is required to thoroughly assess the importance of such a feature.

Quantitative characters are generally less affected by subjective assessment, as the measuring in itself introduces some level of objectivity. However, the usefulness of a quantitative character might easily be overestimated when the variance within individuals and between individuals is not taken into account. Furthermore, quantitative characters can be expected to be influenced by phenotypic plasticity, especially if samples are obtained from varying habitats. For example ‘leaf length’ and ‘leaf width’ would seem to be reasonably different between *R. delavayi* and *R. irroratum* (Fig. 5a and b), as non-overlapping notches might be interpreted as significant difference. However, when taking variation within individuals into account, these two characters are not good choices (Table 3, 1, 2); this also applies to their ratio (Table 3, 11). Even if, in the case of ‘leaf width’, this difference might be significant (highly so if enough samples are used), the high variation within and between individuals would make it impractical to use it for identification of an unknown individual.

Despite the problems highlighted above, several characters were identified that could distinguish between the species. The quantitative characters that showed high discrimination potential ‘flowers in inflorescence’, ‘stigma width’, ‘corolla (width/length)’ and also ‘leaf (dist apex-mid/length)’ (Table 3, 5, 10, 12, 14), tended towards intermediate values for the hybrids (Fig. 5j, l, n, o). As quantitative characters are generally considered to be under polygenic control, this can be expected (Cheverud and Routman 1993).

Many qualitative characters that probably have an underlying quantitative component (bark, corolla colour, indumentum) showed the same picture (Fig. 6b, c and e). Other qualitative characters, on the other hand, either tended towards the morphology seen in one of the parents (stamen hairs were mostly present in the intermediates, as in *R. irroratum*; ovary glands were consistently absent, as in *R. delavayi* (Fig. 6k and g) or showed a combination of intermediate and parental characters (styles were either hairy, or glandular and hairy; and ovaries were either hairy, intermediate, or densely hairy; Fig. 6i and f). The occurrence of both intermediate and parental states in the hybrids for certain characters might well have resulted from the limited number of categories employed, and devising a more graded scale for the hairiness of the ovary (hairs per unit of area) for example, could have shown that none of the hybrids matches the parental species.

While intermediacy is often the expected state in hybrids, it has been shown when averaging over several studies that only about half of the characters in hybrids for interspecific crosses were intermediate, with other characters approaching the morphology of one of the parents (Rieseberg and Ellstrand 1993; Rieseberg and Carney 1998). Obviously this did not apply to the characters chosen in this study, perhaps due to the quantitative nature of many, but further emphasises the need for an adequate number of distinguishing characters, especially as hybrids showed a large variance and some individuals exhibited unusual combinations of character states. However, single individuals only ever exhibited few unusual states, and the MFA provides evidence that the 13 characters used provided enough information to classify even those individuals as intermediate.

With regard to the described varieties and ‘*R. agastum*’ it can be concluded that employing a large enough number of distinguishing characters, and considering hybridization, the intermediate state of most individuals could be identified, even without genetic data available.

#### Described varieties

One issue arising from hybridization is that spontaneous hybrids can be described as species, as was the case with ‘*R. agastum*’. This undoubtedly has the biggest impact on taxonomy, but describing spontaneous hybrids as varieties should be discouraged as well. Although species are often seen by taxonomists as the most important evolutionary entities, this view is probably too simplistic in closely related species complexes; as Darwin already noted that there is an evolutionary continuum between varieties and species (Darwin, 1859, p. 390). This implies that taxa described as varieties (or subspecies) should be groups of individuals that possess evolutionary relevant properties distinguishing them consistently from other members of the species. Such properties can, for example, include a distinct genepool or adaptation to a different habitat. Furthermore, the group should already share a common evolutionary history or have the potential to diverge. Hybrids growing in sympatry with their parents cannot necessarily be assumed to have a distinct genepool, and even if certain hybrids show higher fitness than the parents, it needs to be demonstrated that they have the potential to evolve into a coherent group under natural conditions. Before evidence has been collected in this regard, hybrids should rather be recorded as such rather than described as a taxon.

Together, the two species investigated here have nine described infraspecific taxa: five for *R. delavayi* (var. *delavayi*, var. *adenostylum*, var. *albotomentosum*, var. *peramoenum*, var. *pilostylum*), and four for *R. irroratum*, however, these are all assigned the rank of subspecies, a fact we will not further discuss (subsp. *irroratum*, subsp. *kontumense*, subsp. *pogonostylum*, subsp. *yiliangense*). *R. delavayi* var. *albotomentosum* is described from Myanmar, *R. irroratum* subsp. *kontumense* from Vietnam, and *R. irroratum* subsp. *yiliangense* from a plant in cultivation in Lancaster (UK); these will not be further discussed, as their distribution does not overlap with the study area.

*Rhododendron* *delavayi* var. *delavayi* and *R. irroratum* subsp. *irroratum* correspond to how we would designate the species called *R. delavayi* and *R. irroratum* in this study. All of the infra-specific taxa are mentioned to have a distribution around East Yunnan to West Guizhou, which overlaps with the locality of Baili, and over the whole range it can be assumed that both species *R. delavayi* and *R. irroratum* occur.

*Rhododendron* *delavayi* var. *peramoenum*—this taxon is distinguished from var. *delavayi* largely on the basis of leaf shape and slightly different, thinner indumentum (Chamberlain 1982). As our data suggest, leaf characters do not seem to be consistent enough to unequivocally distinguish the species, which makes a variety described using these characters doubtful. Furthermore, a thinner indumentum would be indicative of individuals of hybrid origin (in this study ‘intermediate’).

*Rhododendron* *delavayi* var. *pilostylum*—this taxon differs from var. delavayi in having a style floccose to the tip (Feng 1983). Although style hair-coverage was polymorphic in *R. delavayi* (Fig. 6j), presence of hairs to the top was only observed in *R. irroratum*. Additionally, style hair-coverage was significantly correlated with the hybrid index, suggesting potential introgression of this character into *R. delavayi* in Baili. Even if it would be polymorphic in ‘pure’ *R. delavayi*, the description of a variety distinguished by this character, as opposed to amending the species description, is of doubtful value.

*Rhododendron* *delavayi* var*. adenostylum*—this taxon is a relatively recently published variety, and was described from Baili from a single herbarium specimen on the basis of a glandular style (Chen *et al.* 2010), presents a case where the character combination used to distinguish it from var. *delavayi* was not observed in intermediates included in the present study. The variety was distinguished by red flower colour (as in *R. delavayi*) but having an entirely glandular style. Purely glandular styles were, however, strongly associated with *R. irroratum* (Fig. 6i), and never observed in combination with red flowers.

Although a purely glandular style cannot be considered transgressive, as it occurs in *R. irroratum*, it showcases a non-intermediate character combination, as can be expected for transgressive individuals. That the plant in question was likely to be uncommon in the population was also recognized by the authors ‘*Rhododendron delavayi* var. *adenostylum* […] is quite rare or might even be destroyed from the wild, because we have not found it again in the area since the original collection was made’ (Chen *et al.* 2010).

*Rhododendron* *irroratum* subsp. *pogonostylum*—this taxon was described as differing from subsp. *irroratum* in having a style that is tomentose and glandular hairy, and a corolla colour that ranges from cream to deep-pink (Chamberlain 1982). In our data, the combination of tomentose and glandular hairs was strongly associated with intermediate individuals, and the type specimen was clearly placed with those (Fig. 6a and i). While the type had a pink corolla, Chamberlain (1982) also mentions white to cream corollas, conforming to individuals likely to be backcrosses close to *R. irroratum* in our data (Fig. 6, around co-ordinates −2, 1), which show an individual with a cream corolla and both tomentose and glandular hairs on the style.

Most of the infra-specific taxa have records from several localities, but as these are based on specimens, it is not possible to ascertain their abundance. Interestingly, there is no clear geographical separation between these taxa, so that rarer varieties or subspecies of the same species seem to always co-occur with var. *delavayi* or subsp. *irroratum*. As *R. delavayi* and *R. irroratum* frequently grow sympatrically throughout their distributions, it can be expected that similar types of hybrid morphologies will also be present in several disjunct areas. Although the data from the single hybrid swarm investigated in this study do not provide enough information to thoroughly re-assess the infraspecific taxa discussed, it is evident that they would fit hybrid morphologies observed at Baili.

Additionally, our data showcase that thorough investigation of a wild population of a potentially new taxon can provide the means to help in the choice of distinguishing characters, and aid in assessing information often not visible from specimen records, such as abundance of the plants in question, and consistency of their morphology.

In many settings early generation hybrids will be more frequent than later generation hybrids, and hence several individuals can be expected to show a similar morphology, perhaps suggesting a sizeable population of the putative new taxon. If, instead of very few characters, a suite of distinguishing traits is used, such cases should be detectable, as a large enough number of the characters can be expected to tend towards intermediacy. Thus, when using a suitable number of morphological traits, if a significant proportion tend to lie between those of sympatric species it should warrant caution. This observation might seem too obvious, but intermediacy can only be assessed if related species growing in sympatry are being considered, which requires information from field observations that is often lacking if descriptions are only based on herbarium specimens.

A further problem can arise from transgressive characters, meaning traits that are not seen in either parent (Rieseberg *et al.* 1999). Transgressive characters can already occur at a lower frequency in F1 hybrids, but are frequently reported in later generation hybrids (Rieseberg and Carney 1998; Hegarty *et al.* 2008; Yakimowski and Rieseberg 2014). If only very few characters are being used to circumscribe a new taxon transgressive characters are necessarily problematic, especially if hybridization is probable. However, as a certain transgressive trait is likely to occur only for certain genomic combinations, it can usually be expected to be rather rare, and therefore an assessment of a certain character in the population in question can yield valuable information.

One case in our study which could be interpreted as a transgressive trait was observed in four intermediate individuals that had significantly shorter styles than either parent. In both species the styles were always longer than the anthers; in the four individuals in question the style was, however, consistently shorter. Not taking other characters into account, for which these individuals were clearly intermediate, one might misinterpret this as a distinguishing trait. In addition to being detectable when using a suite of characters, such cases are more likely to be detected if the population frequency of the trait in question is being taken into consideration.

For over a century taxonomists have been aware of the problems that result from purely specimen-based taxon descriptions (Frodin 2004). As demonstrated above, population based information is often crucial to determine the validity of characters used to distinguish a new taxon, as well as ascertain the uniqueness and consistency of a potentially new taxon. Difficult morphologies that require more taxonomic circumspection can arise with particular ease in large genera in which hybridization occurs, or in geographically widespread groups that show high phenotypic plasticity. Despite many taxonomists being aware of these problems, taxa continue to be published based on few morphological characters, from single or very few specimens and often lacking variability information from natural populations, or based on few individuals with intermediate morphology from a population where both extremes of morphology are present. As was aptly pointed out by Dubois (2011) ‘The time of typological taxonomy is over. It is impossible to properly study, characterise and describe a species on the basis of a single or just a few specimens’. For closely related species and in species complexes, the distinction between a variety or subspecies and a species can often be a philosophical issue, and as such a similar scrutiny should therefore be applied when considering new taxa at these levels.

The taxonomist studying a group in which previously unknown morphologies occur is in the best position to determine the underlying reasons for this, and before a new taxon is defined should have considered other possibilities. Although it might often not be possible to come to a certain conclusion, it should be kept in mind that a post-description assessment will often be more difficult. In large genera, it will be nearly impossible for the revising taxonomist to have good knowledge of natural populations of all the involved species.

In the case of hybridization not only would a higher complexity of names be introduced, but the chance to identify natural processes occurring in the taxa involved would be overlooked. Hybridization is becoming more and more accepted as an integral part of evolution (Abbott *et al.* 2008, 2013), and can indicate a multitude of ongoing processes, including second contact after range expansions (Taylor and Keller 2007; Hewitt 2011), habitat disturbance (Anderson 1948; Bleeker and Hurka 2001; Mitsui *et al.* 2011), or shifts in ecological pressures (Fernandez-Manjarres *et al.* 2006; Buggs and Pannell 2007; Sexton *et al.* 2011). Furthermore, geneflow between species has the potential for a variety of outcomes, from neutral introgression, through transfer of adaptive traits (adaptive introgression), to hybrid speciation (Seehausen 2004; Mallet 2005; Yakimowski and Rieseberg 2014); however, threat to certain species has also been reported (Levin *et al.* 1996).

Hence it is valuable to identify species that are hybridizing, and to record the extent of hybridization in taxonomic groups together with assessing the conditions for which it happens. All this information is lost if hybrids are merely described as varieties or species.

Furthermore, hybrid individuals described as varieties or species will often be uncommon, and the population size of the hypothesized taxon will be estimated to be relatively small. Therefore, such described taxa will often be additionally designated the IUCN status of vulnerable or endangered based on small population size (IUCN 2014). For example in The Red List of *Rhododendrons* (Gibbs *et al.* 2011) 25 taxa that are listed with status vulnerable, endangered or critically endangered are explicitly mentioned to pose taxonomic problems, often related to hybridization or uncertain variety descriptions. It is important to point out that these are only the taxa assigned a rank, as the authors seem to have taken a conservative approach listing numerous further taxa as data deficient. This can in the worst case lead to misdirected conservation effort, as was reported to have occurred with the orchid *Dactylorhiza lapponica* in the UK (Pillon and Chase 2007).

## Conclusions

It is nowadays evident that hybridization is widespread in certain groups of organisms, and the boundaries between varieties and species can be blurred in complexes of closely related species. To further our understanding of present day diversity and evolutionary processes it is therefore crucial that taxonomists take a population-based approach to taxonomic treatments. This includes not returning to a purely specimen based approach to systematics, and taking the situation in the field into account when considering new taxa. When new varieties or species are being described, these should be based on an adequate number of characters, which are sufficiently discriminating when the variance within individuals and within populations has been taken into account, implying that the description is based on a group of individuals exhibiting a similar morphology. Furthermore, the authors should have demonstrated that they have considered the possibility of hybridization, and are aware of related species that occur in sympatry with the newly described taxon, especially in species rich genera or when the genus is known to be prone to hybridization.

If the morphology of plants is intermediate between two species with which it is sympatric, we suggest collecting specimens and labelling them as a potential nothotaxon requiring further investigation (e.g. *Rhododendron irroratum* × *delavayi*). In this way, the morphology is recorded but no new taxon is described. Comments regarding species complexes can also be made as part of a revision (see, for example, Chamberlain 1982).

## Sources of Funding

This work was supported by The National Natural Science Foundation of China, Yunnan (grant U1302262 to W.S.); and the Co-operation Project of the Chinese Academy of Sciences and Guizhou Province (grant ‘Sheng Di Yuan He 2011-2’); T.M. was funded by the Yunnan Provincial Government (grants Y53A531261 and Y51I611361).

## Contributions by the Authors

T.M. and J.M. conceived the study, carried out fieldwork, and wrote the manuscript; T.M. performed statistical analysis; Y.M. and X.Z. carried out fieldwork and measurements; W.S. and Y.M. provided funding.

## Conflict of Interest Statement

None declared.

## Supplementary Material

Supplementary Data

## Supporting Information

The following additional information is available in the online version of this article —

**Zip Archive** containing morphological scoring data and R scripts used to carry out analysis.

**PDF** containing information on Examination and measurement of type material and Exploratory MFA.

**Table S1.** Ranges of variances of measured quantitative characters in single individuals.

**Table S2.** Group variances of measured quantitative characters.

**Table S3.** Rhododendron varieties and species with unclear taxonomic status that are mentioned in the red book of rhododendrons (Gibbs et al. 2011).

**Figure S1.** Multiple Factor Analysis (MFA) for individuals from Baili.

**Figure S2.** Eigenvalues of the MFA analysis.

## References

[plw070-B1] Abbott R Albach D Ansell S Arntzen JW Baird SJE Bierne N Boughman J Brelsford A Buerkle CA Buggs R Butlin RK Dieckmann U Eroukhmanoff F Grill A Cahan SH Hermansen JS Hewitt G Hudson AG Jiggins C Jones J Keller B Marczewski T Mallet J Martinez-Rodriguez P Möst M Mullen S Nichols R Nolte AW Parisod C Pfennig K Rice AM Ritchie MG Seifert B Smadja CM Stelkens R Szymura JM Väinölä R Wolf JBW Zinner D. 2013 Hybridization and speciation. Journal of Evolutionary Biology 26:229–246. 23323997 10.1111/j.1420-9101.2012.02599.x

[plw070-B2] Abbott RJ Ritchie MG Hollingsworth PM. 2008 Introduction. Speciation in plants and animals: pattern and process. Philosophical Transactions of the Royal Society B: Biological Sciences 363:2965–2969. 10.1098/rstb.2008.0096 PMC2459220 18583278

[plw070-B3] Anderson E. 1948 Hybridization of the habitat. Evolution 2:1–9.

[plw070-B4] Anderson E Stebbins GL. 1954 Hybridization as an evolutionary stimulus. *Evolution*. 8:378–388.

[plw070-B5] Bardy KE Schönswetter P Schneeweiss GM Fischer MA Albach DC. 2011 Extensive gene flow blurs species boundaries among *Veronica barrelieri*, *V. orchidea* and *V. spicata* (Plantaginaceae) in southeastern Europe. Taxon 60:108–121.

[plw070-B6] Barrington DS. 2011 Should hybrids be protected by listing, *Betula x sandbergii* and *Botrychium minganense* in Vermont. The Journal of the Torrey Botanical Society 138:465–471.

[plw070-B7] Bleeker W Hurka H. 2001 Introgressive hybridization in *Rorippa* (Brassicaceae): gene flow and its consequences in natural and anthropogenic habitats. Molecular Ecology 10:2013–2022. 11555244 10.1046/j.1365-294x.2001.01341.x

[plw070-B8] Buggs RJA Pannell JR. 2007 Ecological differentiation and diploid superiority across a moving ploidy contact zone. Evolution 61:125–140. 17300432 10.1111/j.1558-5646.2007.00010.x

[plw070-B9] Chamberlain DF. 1982 A revision of *Rhododendron*, II. Subgenus Hymenanthes. Notes from the Royal Botanic Garden Edinburgh 39:209–486.

[plw070-B10] Chamberlain DF Hyam R Argent G Fairweather G Walter KS. 1996 The genus rhododendron: its classification and synonymy. Edinburgh: Royal Botanic Garden Edinburgh.

[plw070-B11] Chen X Consaul L Huang J Chen X. 2010 New taxa of *Rhododendron* (Ericaceae) from China. Annales Botanicae Fennici 47:397–402.

[plw070-B12] Cheverud JM Routman E. 1993 Quantitative trait loci: individual gene effects on quantitative characters. Journal of Evolutionary Biology 6:463–480.

[plw070-B13] Cockerham CC. 1973 Analyses of gene frequencies. Genetics 74:679–700. 17248636 10.1093/genetics/74.4.679 PMC1212983

[plw070-B14] Darwin C. 1859 On the origin of species, 1st edn London: John Murray www.gutenberg.org, Ebook #1228 (09 October 2016).

[plw070-B15] Doorenbos J. 1955 Shortening the breeding cycle of *Rhododendron*. Euphytica 4:141–146.

[plw070-B16] Dowell P. 1908 New ferns described as hybrids in the genus *Dryopteris*. Bulletin of the Torrey Botanical Club 35:135–140.

[plw070-B17] Dubois A. 2011 Describing a new species. Taprobanica: The Journal of Asian Biodiversity 2:6–24.

[plw070-B18] Ellstrand NC Whitkus R Rieseberg LH. 1996 Distribution of spontaneous plant hybrids. Proceedings of the National Academy of Sciences USA 93:5090–5093. 10.1073/pnas.93.10.5090 PMC39411 11607681

[plw070-B19] Fang M Fang R He M Hu L Yang H Qin H Tianlu M Chamberlain DF Stevens PF Wallace GD Anderberg A. 2005 *Ericaceae* In: Wu Z Raven PH Hong D, eds. Flora of China: Ericaceae, vol. 14 Beijing/St. Louis: Science Press/Missouri Botanical Garden, 242–517.

[plw070-B20] Feng K. 1983 New species and varieties of *Rhododendron* from Yunnan. Acta Botanica Yunnanica 5:265–270.

[plw070-B21] Fernandez-Manjarres JF Gerard PR Dufour J Raquin C Frascaria-Lacoste N. 2006 Differential patterns of morphological and molecular hybridization between *Fraxinus excelsior* L. and *Fraxinus angustifolia* Vahl (Oleaceae) in eastern and western France. Molecular Ecology 15:3245–3257. 16968268 10.1111/j.1365-294X.2006.02975.x

[plw070-B22] Frodin DG. 2004 History and concepts of big plant genera. Taxon 53:753–776.

[plw070-B23] Gibbs D Chamberlain D Argent G. 2011 The red list of Rhododendrons. Surrey: Botanic Gardens Conservation International.

[plw070-B24] Hegarty MJ Barker GL Brennan AC Edwards KJ Abbott RJ Hiscock SJ. 2008 Changes to gene expression associated with hybrid speciation in plants: further insights from transcriptomic studies in *Senecio*. Philosophical Transactions of the Royal Society B: Biological Sciences 363:3055–3069. 10.1098/rstb.2008.0080 PMC2607317 18579474

[plw070-B25] Hegarty MJ Hiscock SJ. 2005 Hybrid speciation in plants: new insights from molecular studies. New Phytologist 165:411–423. 15720652 10.1111/j.1469-8137.2004.01253.x

[plw070-B26] Hewitt GM. 2011 Quaternary phylogeography: the roots of hybrid zones. Genetica 139:617–638. 21234647 10.1007/s10709-011-9547-3

[plw070-B27] IUCN. 2014. Guidelines for Using the IUCN Red List Categories and Criteria.

[plw070-B56] Version 11. http://www.iucnredlist.org/documents/RedListGuidelines.pdf (27 November 2015).

[plw070-B28] Kron KA. 1997 Phylogenetic relationships of Rhododendroideae (Ericaceae). American Journal of Botany 84:973–980. 21708652

[plw070-B29] Lê S Josse J Husson F. 2008 FactoMineR: an R Package for multivariate analysis. Journal of Statistical Software 25:1–18.

[plw070-B30] Levin DA Francisco-Ortega J Jansen RK. 1996 Hybridization and the extinction of rare plant species. Conservation Biology 10:10–16.

[plw070-B31] Liu Z Chen X Cribb PJ. 2009 *91* In: Wu, Z, Raven, PH, Hong, D. eds. *Cymbidium*, vol. 25. Beijing/St. Louis: Science Press/Missouri Botanical Garden, 260–280.

[plw070-B32] Mallet J. 2005 Hybridization as an invasion of the genome. Trends in Ecology and Evolution 20:229–237. 16701374 10.1016/j.tree.2005.02.010

[plw070-B33] Marczewski T Chamberlain DF Milne RI. 2015 Hybridization in closely related *Rhododendron* species: half of all species-differentiating markers experience serious transmission ratio distortion. Ecology and Evolution 5:3003–3022. 26357534 10.1002/ece3.1570 PMC4559045

[plw070-B34] Milne RI Abbott RJ. 2008 Reproductive isolation among two interfertile *Rhododendron* species: low frequency of post-F1 hybrid genotypes in alpine hybrid zones. Molecular Ecology 17:1108–1121. 18261051 10.1111/j.1365-294X.2007.03643.x

[plw070-B35] Milne RI Abbott RJ Wolff K Chamberlain DF. 1999 Hybridization among sympatric species of *Rhododendron* (Ericaceae) in Turkey: morphological and molecular evidence. American Journal of Botany 86:1776–1785. 10602769

[plw070-B36] Milne RI Davies C Prickett R Inns LH Chamberlain DF. 2010 Phylogeny of *Rhododendron* subgenus *Hymenanthes* based on chloroplast DNA markers: between-lineage hybridisation during adaptive radiation? Plant Systematics and Evolution 285:233–244.

[plw070-B37] Milne RI Terzioglu S Abbott RJ. 2003 A hybrid zone dominated by fertile F1s: Maintenance of species barriers in *Rhododendron*. Molecular Ecology 12:2719–2729. 12969475 10.1046/j.1365-294x.2003.01942.x

[plw070-B38] Ming T. 1984 A revision of subgenus *Hymenanthes* (*Rhododendron* L.) in Yunnan-Xizang. Acta Botanica Yunnanica 6:141–171.

[plw070-B39] Mitsui Y Nomura N Isagi Y Tobe H Setoguchi H. 2011 Ecological barriers to gene flow between riparian and forest species of *Ainsliaea* (Asteraceae). Evolution 65:335–349. 20840597 10.1111/j.1558-5646.2010.01129.x

[plw070-B40] Parnell JAN Pedersen H&AElig; Hodkinson TR Balslev H van Welzen PC Simpson D Middleton DJ Esser HJ Pooma R Utteridge T Staples G. 2013 Hybrids and the Flora of Thailand. Thai Forrest Bulletin (Botany) 41:1–9.

[plw070-B41] Pillon Y Chase MW. 2007 Taxonomic exaggeration and its effects on orchid conservation. Conservation Biology 21:263–265. 17298532 10.1111/j.1523-1739.2006.00573.x

[plw070-B42] R Development Core Team. 2016 R: A Language and Environment for Statistical Computing. (https://www.R-project.org/).

[plw070-B43] Rieseberg LH. 1997 Hybrid origins of plant species. Annual Review of Ecology and Systematics 28:359–389.

[plw070-B44] Rieseberg LH Archer MA Wayne RK. 1999 Transgressive segregation, adaptation and speciation. Heredity 83:363–372. 10583537 10.1038/sj.hdy.6886170

[plw070-B45] Rieseberg LH Carney SE. 1998 Tansley review No. 102 plant hybridization. New Phytologist 140:599–624. 10.1046/j.1469-8137.1998.00315.x 33862960

[plw070-B46] Rieseberg LH Ellstrand NC. 1993 What can molecular and morphological markers tell us about plant hybridization? Critical Reviews in Plant Sciences 12:213–241.

[plw070-B47] Rieseberg LH Willis JH. 2007 Plant speciation. Science 317:910–914. 17702935 10.1126/science.1137729 PMC2442920

[plw070-B48] Seehausen O. 2004 Hybridization and adaptive radiation. Trends in Ecology and Evolution 19:198–207. 16701254 10.1016/j.tree.2004.01.003

[plw070-B49] Sexton JP Strauss SY Rice KJ. 2011 Gene flow increases fitness at the warm edge of a species’ range. Proceedings of the National Academy of Sciences 108:11704–11709. 10.1073/pnas.1100404108 PMC3136252 21709253

[plw070-B50] Taylor DR Keller SR. 2007 Historical range expansion determines the phylogenetic diversity introduced during contemporary species invasion. Evolution 61:334–345. 17348944 10.1111/j.1558-5646.2007.00037.x

[plw070-B51] Whitney KD Ahern JR Campbell LG Albert LP King MS. 2010 Patterns of hybridization in plants. Perspectives in Plant Ecology, Evolution and Systematics 12:175–182.

[plw070-B52] Yakimowski SB Rieseberg LH. 2014 The role of homoploid hybridization in evolution: a century of studies synthesizing genetics and ecology. American Journal of Botany 101:1247–1258. 25156978 10.3732/ajb.1400201

[plw070-B53] Zha HG Milne RI Sun H. 2008 Morphological and molecular evidence of natural hybridization between two distantly related *Rhododendron* species from the Sino-Himalaya. Botanical Journal of the Linnean Society 156:119–129.

[plw070-B54] Zha HG Milne RI Sun H. 2010 Asymmetric hybridization in *Rhododendron agastum*: a hybrid taxon comprising mainly F1s in Yunnan, China. Annals of Botany 105:89–100. 19887474 10.1093/aob/mcp267 PMC2794068

[plw070-B55] Zhang JL Zhang CQ Gao LM Yang JB Li HT. 2007 Natural hybridization origin of *Rhododendron agastum* (Ericaceae) in Yunnan, China: inferred from morphological and molecular evidence. Journal of Plant Research 120:457–463. 17393071 10.1007/s10265-007-0076-1

