# Supplementary material for: Why is population information crucial for taxonomy? A case study involving a hybrid swarm and related varieties
Source: AoB Plants. 2016 Nov 11;8:plw070. doi: 10.1093/aobpla/plw070 (PMC5142052; doi:10.1093/aobpla/plw070)
Supplement: Supplementary Data [file supp_8_plw070_index.html]

Why is population information crucial for taxonomy? A case study involving a hybrid swarm and related varieties — Supplementary Data 

# Why is population information crucial for taxonomy? A case study involving a hybrid swarm and related varieties

## Supplementary Data

files

- Supplementary Data - zip file
