# Supplementary material for: Why is population information crucial for taxonomy? A case study involving a hybrid swarm and related varieties
Source: AoB Plants. 2016 Nov 11;8:plw070. doi: 10.1093/aobpla/plw070 (PMC5142052; doi:10.1093/aobpla/plw070)
Supplement: Supplementary Data [file supp_plw070_suppl_data.zip › Supporting_Information.pdf]

SUPPORTING INFORMATION

Why is population information crucial for taxonomy? A case study involving a hybrid swarm and related varieties

(Marczewski et al. 2016)

Examination and measurement of type material

Type specimens of *R. delavayi* var. *adenostylum* (Y.K. Li 11679, holo HGAS) and *R. delavayi* var. *pilostylum* (C.W. Wang 87289, holo KM) were examined and, where possible, measurements were obtained as for the Baili individuals. For the types of *R. delavayi* var. *peramoenum* (Forrest 17708, holo E iso K), *R. irroratum* var. *pogonostylum* (Henry 11066, holo K iso E), and *R. agastum* (Forrest 9920, holo E) only high-resolution scanned images from both E (http://elmer.rbge.org.uk/bgbase/vherb/bgbasevherb.php - specimen barcodes E00001402, E00001039, E00010358, and E00001029) and K (http://apps.kew.org/herbcat/gotoSearchPage.do - specimen barcodes K000769484 and K000769527) were downloaded. Measurements were then obtained using the software GIMP (http://www.gimp.org/) with the “measure” tool and later converted from pixels to metric units.

Many of the measurements taken from living plants were not available when examining type material, and in no case did the specimens have the required number of leaves or flowers (10) displayed in a way that allowed measurement. Bark characters were not assessable at all because the specimens examined comprised only small branches bearing one or few inflorescences, and in no case did the collector note the morphology of the bark. Maculation was not visible on all specimens, and the distance between anther and style was impossible to measure because in all cases the flowers had fallen apart. The colour of the style was also impossible to assess on dried material. Stigma width was found to differ substantially depending on whether the measurements were taken from a fresh flower or a dried and pressed one, and was therefore excluded from our analyses.

The number of flowers per inflorescence was inferred from the specimen. To obtain an estimate visible flowers, ovaries and pedicel scars were counted and additionally the number of flowers not directly visible (on the back of the

inflorescence) was extrapolated accordingly. In all cases, corolla colour was taken from specimen label notes.

The two specimens to which we had direct access were examined for hairs on stamens, ovaries and styles employing a handlens. Due to the age of the specimens (some >70 years old) it is possible that hairs have been rubbed off. Hairiness was not always unequivocally visible on the scanned specimens. Therefore we mostly relied on previous descriptions (Chamberlain 1982) and collector's notes. In two cases (Henry 11066 and Forrest 17708) the collector's notes mention stamen hairs, and all three mention hairiness of the ovary and style, but do not explicitly mention glands. However, that glandular hairs are present on the ovary in Forrest 9920 and Henry 11066 is stated in a revision of Rhododendron (Chamberlain, 1982, pages 301 and 296-298 respectively). Although *R. delavayi* var. *adenostylum* was described on the basis of a glandular style, all styles we assessed on the type seemed glabrous. Because of this, included this specimen in our analyses twice, once based on our observations and once based on the description.

Exploratory MFA

Initially an MFA was performed including all scored characters, apart from “stamen hairs” and “style hair-coverage” (Table 1; 17, 26), as these were the characters that had been used to describe the varieties discussed later, and therefore it was desired that they not have any impact on the groupings. For the analysis the option of scaling quantitative characters to unit variance was used. To reduce noise in the analysis, characters which were not represented well by the first two dimensions (as assessed by how much of their variation was explained by one of the axes), were then removed. Furthermore, “style colour” was removed, as it was significantly correlated with corolla colour.

Table S1. Ranges of variances of measured quantitative characters in single individuals.

Range of variances observed in individuals, based on ten repeated measurements per individual. Shown are the ranges for the three investigated groups, *R. delavayi*, *R. irroratum*, and intermediates. For each character, the equality of variances was tested with a Brown-Forsythe Test (B-F), and a significant p-value indicates that the variances in the respective group can not be assumed to be all equal.

**Character**	**Group**					
	R. delavayi		**intermediate**		***R. irroratum***	
	**range**	**B-F p-value**	**range**	**B-F p-value**	**range**	**B-F p-value**
leaf length	1.634 - 16.242	0.15	1.072 - 15.037	0.11	0.502 - 8.244	0.01
leaf width	11.225 - 159.483	0.31	6.933 - 112.166	0.01	3.878 - 81.965	0.02
distance apex-mid	47.749 - 315.309	0.34	15.567 - 298.728	0	18.481 - 170.722	0.02
petiole length	5.052 - 48.545	0.02	2.272 - 62.933	0	1.433 - 24.322	0.01
flowers in inflorescence	1.700 - 33.944	0.07	0.844 - 34.528	0.01	0.839 - 10.989	0.21
corolla width	0.034 - 0.472	0.09	0.017 - 0.529	0	0.009 - 0.751	0
corolla length	0.051 - 0.458	0.26	0.018 - 0.773	0.03	0.010 - 0.512	0
distance anther-stigma	1.192 - 26.250	0.02	0 - 43.092	0	0.611 - 14.361	0
style length	0.023 - 0.430	0.03	0.011 - 0.979	0	0.005 - 0.396	0
stigma width	0.021 - 0.208	0.82	0.002 - 0.186	0	0.021 - 0.180	0.03
leaf (width / length)	<0.001 - 0.004	0.04	<0.001 - 0.005	0.01	<0.001 - 0.004	0.05
leaf (dist apex-mid / length)	<0.001 - 0.006	0.34	<0.001 - 0.009	0.51	<0.001 - 0.003	0.34
leaf (petiole / length)	<0.001 - 0.003	0.03	<0.001 - 0.002	0	<0.001 - 0.001	0.15
corolla (width / length)	<0.001 - 0.017	0.05	<0.001 - 0.044	0	<0.001 - 0.012	0.3

B-F = Brown-Forsythe test p-value

Table S2. Group variances of measured quantitative characters.

Group variances of quantitative characters based on trimmed means for each individual (the two smallest and two largest measurements were removed - each individual mean is based on six measurements). Shown is the variance observed in each of the three groups, *R. delavayi*, *R. irroratum*, and intermediates. A Brown-Forsythe Test (B-F) was used to test for equality of variances, once including all three groups (all), and once comparing only the parental species, *R. delavayi* and *R. irroratum* (par).

**Character**	**Group**			**B-F p-value**	
	**R. delavayi**	**intermediate**	**R. irroratum**	**all**	**par**
leaf length	1.525	1.344	1.942	0.697	0.524
leaf width	9.141	10.531	11.699	0.983	0.874
distance apex-mid	34.597	29.333	46.766	0.422	0.579
petiole length	7.900	5.474	4.735	0.411	0.225
flowers in inflorescence	8.163	7.488	4.994	0.664	0.407
corolla width	0.181	0.254	0.149	0.286	0.636
corolla length	0.190	0.294	0.278	0.394	0.194
distance anther-stigma	8.725	10.623	7.136	0.525	0.682
style length	0.242	0.256	0.214	0.892	0.794
stigma width	0.120	0.127	0.064	0.277	0.115
leaf (width / length)	0.001	0.001	0.001	0.296	0.905
leaf (dist apex-mid / length)	0.001	0.001	0.001	0.900	0.632
leaf (petiole / length)	0.000	0.000	0.000	0.840	0.725
corolla (width / length)	0.002	0.005	0.006	0.152	0.046

B-F = Brown-Forsythe test p-value
All = all three groups were used for the B-F test.
Par = only the two parents (R. delavayi and R. irroratum) were used to carry out the B-F test.

Table S3. Rhododendron varieties and species with unclear taxonomic status that are mentioned in the Red Book of Rhododendrons (Gibbs et al. 2011).

Only taxa that have not been assigned category DD (data deficient) are listed. The “Notes” field mentions the wording that the assessment of the unclear status is based on. Of the listed taxa 19 were assigned the status vulnerable (VU); 4 endangered (EN); and 2 critically endangered (CR).

Species / Variety	Status	Distribution	Notes
R. amesiae	CR B1	China (Sichuan)	[...] Some debate exists around the taxonomic status and wether its characteristics are significant enough to warrant its distinction from R. concinnum.
R. argyrophyllum subsp. omeiense	VU D2	China (Sichuan)	Debate still exists over its taxonomic status
R. calophytum var. jinfuense	VU D1	China (Chongqing)	[...] there is debate about the status of this taxon as a separate variety.
R. calvescens	VU B1	China (Xizang, Yunnan)	[...] Some believe it to be a natural hybrid [...]
R. chamaethomsonii var. chamaedoron	VU B1	China (Xizang, Yunnan)	[...] Taxonomic debate exists with some believing it to be a form of R. forrestii and a natural hybrid
R. chamaethomsonii var. chamaethauma	VU D2	China (Xizang, Yunnan)	[...] Taxonomic debate exists with some believing it to be a form of R. forrestii and a natural hybrid
R. clementinae subsp. aureodorsale	VU D2	China (Shaanxi)	[...] Some debate exists around the taxonomic status of the taxon, considered either a species in its own right or a subspecies of R. przewalskii.
R. codonanthum	VU B2	China (Yunnan)	[...] some believe it to be a natural hybrid of R. proteoides x R. sanguineum.
R. comisetum	VU D2	China (Xizang, Yunnan)	[...] possibly a hybrid between R. proteoides and R. temenium
R. erastum	VU D2	China (Xizang, Yunnan)	[...] potentially a natural hybrid
R. esetulosum	VU D2	China (Xizang, Yunnan)	[...] Considered by some to be a natural hybrid of R. selense
R. eurysiphon	EN B1	China (Xizang), Myanmar	[...] Some taxonomists believe that this is a natural hybrid.
R. forrestii subsp. papillatum	VU D2	China (Xizang)	[...] Some debate exists around the taxonomic status of this subspecies.
R. guangnanense	CR B1+2	China (Yunnan)	[...] Some debate exists about its taxonomic status.
R. longicalyx	VU D2	China (Sichuan)	[...] Some taxonomists consider this to be a variety of R. souliei
R. mackenzianum	VU D1+2	China (Xizang, Yunnan), Myanmar	[...] Some taxonomic debate about its status exists.
R. nakaharae	EN B1	Taiwan	[...] Considered by some to be a naturally occurring hybrid
R. neriiflorum var. agetum	VU D2	China (Yunnan)	[...] Possibly a synonym of R. neriiflorum var. neriiflorum
R. petrocharis	VU D2	China (Guizhou, Sichuan)	[...] Some taxonomic debate about its status exists.
R. platypodum	EN B1	China (Chongqing)	[...] Some debate exists about whether or not a closely related taxon, R. yuefengense is synonymous with this species.
R. pubicostatum	EN B1	China (Yunnan)	[...] Some taxonomic debate exists around whether or not this is a natural hybrid.
R. rex subsp. gratum	VU D2	China (Yunnan)	[...] Considered a synonym of R. basilicum by some taxonomists
R. sinonuttallii	VU D2	China (Xizang, Yunnan), Myanmar	[...] Taxonomically uncertain and considered by some to be close to R. nuttallii
R. wattii	VU D2	India (Manipur)	[...] Debate remains as to whether this is a good species or a natural hybrid between R. macabeanum and R. arboreum subsp. delavayi.
R. websterianum var. yulongense	VU D2	China (Sichuan)	[...] some taxonomic debate exists about whether the differences are significant enough to justify subspecies classification.

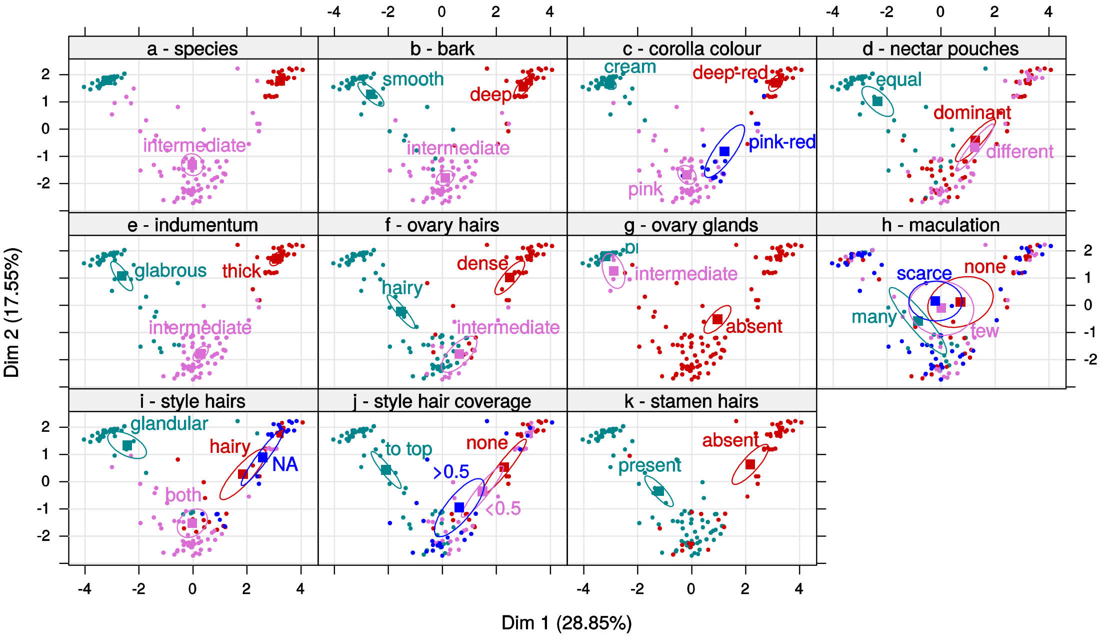

Figure S1. Multiple Factor Analysis (MFA) for individuals from Baili. Results from a MFA carried out using 13 characters (see Table 1). Following characters shown here: species (a), maculation (h), style hair-coverage (j), and stamen hairs (k) were not used to calculate the MFA and were only plotted onto the coordinates for graphical assessment.

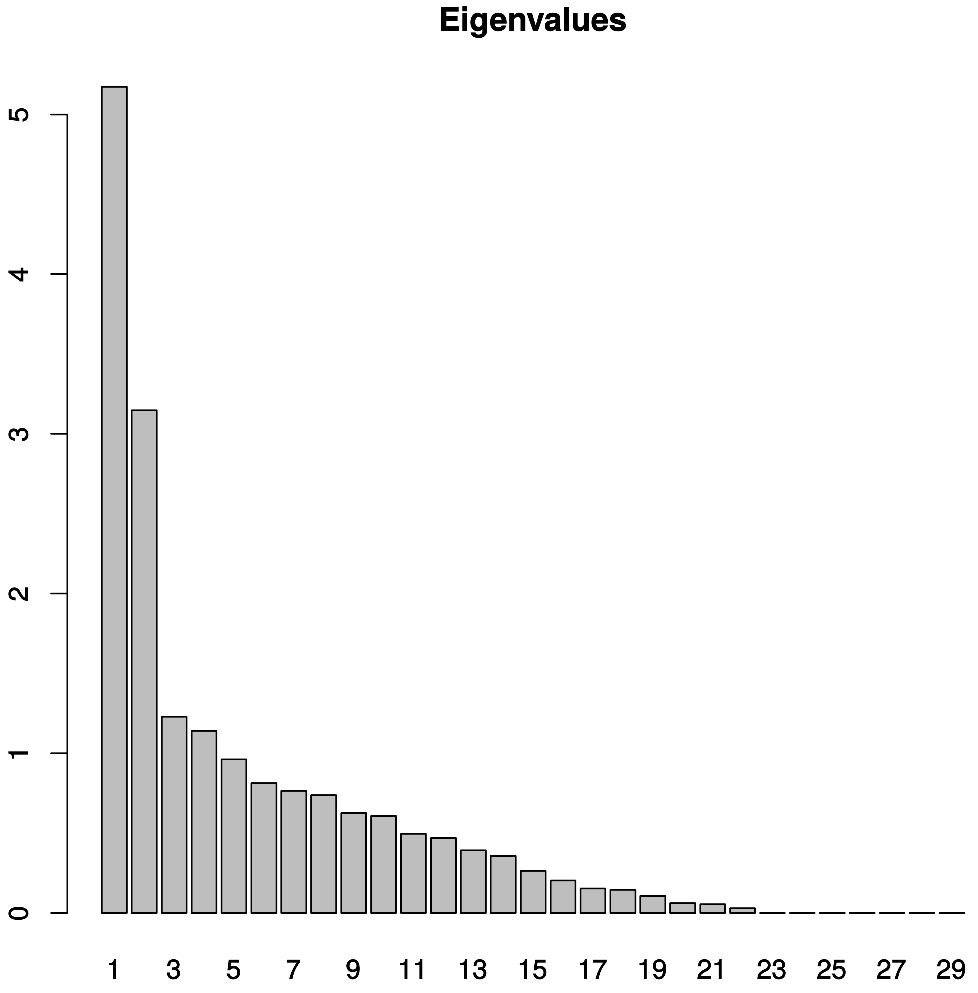

Figure S2. Eigenvalues of the MFA analysis. The eigenvalues returned by the MFA analysis indicate that the first two axes are significantly larger than the others (employing the criterion of a scree plot). Hence a two-dimensional representation is adequate to represent the data.
